# Effect of Disease Severity, Age of Child, and Clinic No-Shows on Unscheduled Healthcare Use for Childhood Asthma at an Academic Medical Center

**DOI:** 10.3390/ijerph20021508

**Published:** 2023-01-13

**Authors:** Pavani Rangachari, Imran Parvez, Audrey-Ann LaFontaine, Christopher Mejias, Fahim Thawer, Jie Chen, Niharika Pathak, Renuka Mehta

**Affiliations:** 1 Department of Population Health & Leadership, School of Health Sciences, University of New Haven, West Haven, CT 06516, USA; 2 Division of Biostatistics and Data Science, Department of Population Health Sciences, Medical College of Georgia, Augusta University, Augusta, GA 30912, USA; 3 Medical College of Georgia, Augusta University, Augusta, GA 30912, USA; 4 Division of Critical Care Medicine, Department of Pediatrics, Medical College of Georgia, Augusta University, Augusta, GA 30912, USA

**Keywords:** pediatric asthma, supported self-management of asthma, asthma severity, clinic no-shows, unscheduled healthcare use, social determinants of health

## Abstract

This study examines the influence of various individual demographic and risk factors on the use of unscheduled healthcare (emergency and inpatient visits) among pediatric outpatients with asthma over three retrospective timeframes (12, 18, and 24 months) at an academic health center. Out of a total of 410 children who visited an academic medical center for asthma outpatient care between 2019 and 2020, 105 (26%) were users of unscheduled healthcare for childhood asthma over the prior 12 months, 131 (32%) over the prior 18 months, and 147 (36%) over the prior 24 months. multiple logistic regression (MLR) analysis of the effect of individual risk factors revealed that asthma severity, age of child, and clinic no-shows were statistically significant predictors of unscheduled healthcare use for childhood asthma. Children with higher levels of asthma severity were significantly more likely to use unscheduled healthcare (compared to children with lower levels of asthma severity) across all three timeframes. Likewise, children with three to four clinic no-shows were significantly more likely to use unscheduled healthcare compared to children with zero clinic no-shows in the short term (12 and 18 months). In contrast, older children were significantly less likely to use unscheduled healthcare use compared to younger children in the longer term (24 months). By virtue of its scope and design, this study provides a foundation for addressing a need identified in the literature for short- and long-term strategies for improving supported self-management and reducing unscheduled healthcare use for childhood asthma at the patient, provider, and organizational levels, e.g., (1) implementing telehealth services for asthma outpatient care to reduce clinic no-shows across all levels of asthma severity in the short term; (2) developing a provider–patient partnership to enable patient-centered asthma control among younger children with higher asthma severity in the long term; and (3) identifying hospital–community linkages to address social risk factors influencing clinic no-shows and unscheduled healthcare use among younger children with higher asthma severity in the long term.

## 1. Introduction

Asthma is the most common chronic condition among children less than 18 years of age, with more than 10 million children in the US estimated to have an asthma diagnosis [1]. Nearly 5 million children experience an asthma exacerbation each year in the US. Acute exacerbation of asthma symptoms often requires unscheduled healthcare use, including emergency department (ED) visits and hospitalizations, which contributes to over half of annual expenditures for pediatric asthma, with increasing hospital charges over time [1,2,3].

Both the National Heart Lung and Blood Institute (NHLBI) guidelines and recent meta-reviews of the literature on asthma management have emphasized that unscheduled healthcare use for asthma can be prevented through “supported self-management of asthma”, which refers to a provider–patient partnership to empower patients to regularly monitor asthma symptoms, make necessary treatment adjustments, and take control of their disease [4]. To this end, research has underscored the need for active provider and organizational (hospital/clinic) engagement in asthma management. However, substantial gaps remain in integrating supported self-management of asthma into routine clinical practice across the United States [5,6,7,8,9].

AU Health, a tertiary care academic medical center based in Augusta, Georgia, USA, was no different in experiencing the challenge of unscheduled healthcare use for childhood asthma and limited engagement of providers in improving the self-management effectiveness (SME) of childhood asthma. For example, out of 410 unique children who visited AU Health pediatric outpatient clinics for asthma between 1 January 2019, and 31 December 2020, 131 were users of unscheduled healthcare for asthma over a previous 18-month timeframe. In other words, 32% of these children either visited the ED or were hospitalized at least once over the previous 18 months for asthma. When these data were broken down by disease (asthma) severity, we found that of the 131 users of unscheduled healthcare, only 14 (11%) had severe-persistent asthma, 60 (45%) had moderate-persistent asthma, and the remaining 57 (44%) had mild or intermittent asthma. Importantly, there were non-users of unscheduled healthcare (i.e., children who had zero ED visits and zero hospitalizations over the previous 18-month timeframe) in every asthma severity category, including severe-persistent, moderate-persistent mild-persistent, and intermittent asthma. In other words, although asthma severity was clearly an important predictor of unscheduled healthcare use, it did not fully explain unscheduled healthcare use since there were users and non-users of unscheduled healthcare across all asthma severity categories. This implied that there were other factors explaining unscheduled healthcare use for childhood asthma, which in turn indicated the potential for mitigating this problem through better management of asthma, including provider engagement in supported self-management of childhood asthma.

Existing research on asthma management has emphasized that a key pre-requisite for provider engagement in improving SME of childhood asthma is the availability of evidence regarding what drives unscheduled healthcare use among patients [10,11]. This information in turn is essential for understanding key predictors of unscheduled healthcare use and for developing a foundation to identify effective strategies and interventions for reducing the burden of unscheduled healthcare use for childhood asthma. In keeping with this rationale, the primary purpose of this retrospective study was to identify the significant predictors (risk factors) for unscheduled healthcare use among pediatric outpatients with asthma at the study institution (AU Health). 

### 1.1. Literature Review and Conceptual Framework

Existing studies on factors influencing self-management effectiveness (SME) and unscheduled healthcare use for childhood asthma have highlighted the role of multiple determinants of childhood asthma SME. Importantly, the literature has emphasized that unscheduled healthcare use for childhood asthma could serve as a primary outcome measure for childhood asthma SME [4,5,6,7,8,9,10,11]. With respect to the multiple determinants of childhood asthma SME, the literature has put forth a “holistic framework” for measuring childhood asthma SME that calls for the consideration of multiple levels, including, individual, interpersonal, organizational (health system), community, and environmental levels of influence on childhood asthma SME. 

The following provide some examples under each category: (1) Individual risk factors would include demographic characteristics, such as age, gender, race, and insurance; biologic risk factors, such as disease or asthma severity and Body-Mass Index (BMI); and behavioral risk factors such as clinic no-shows, clinic cancellations, smoking, medication adherence, and symptom control. (2) Interpersonal risk factors would include the social support network for asthma control. (3) Organizational- or health system-level factors would include the quality of provider–patient communication on asthma management. (4) Community-level risk factors would include financial hardship, access to asthma care, food, transportation, and housing stability. Lastly, (5) environmental risk factors would include factors such as outdoor temperature, heat, and outdoor and indoor air quality [10,11,12,13]. According to the “holistic framework” for assessing asthma SME, each of these factors (separately and together) can impact SME in childhood asthma, with SME defined by the primary outcome of unscheduled healthcare use for childhood asthma [10,11].

### 1.2. Study Purpose and Significance

This study seeks to address the following research question: “What are the individual risk factor differences between users and non-users of unscheduled healthcare among pediatric outpatients with asthma at an academic medical center?” The study, in turn, was guided by the “holistic framework” for assessing asthma SME (discussed above). However, since the study relies on a retrospective review of medical records, it was focused on examining the relationship between specific individual risk factors (outlined below) and unscheduled healthcare use for childhood asthma. The individual risk factors of interest were: (1) asthma severity, (2) age, (3) gender, (4) race, (5) insurance, (6) Body-Mass Index (BMI), (7) medication adherence, (8) smoking, (9) no-shows for asthma clinic visits; and (10) cancellations of asthma clinic visits. 

Despite a focus on individual risk factors, it would be relevant to note that this study is designed to examine the concurrent influence of individual biologic, demographic, and behavioral risk factors (e.g., asthma severity, age, and clinic no-shows) on unscheduled healthcare within the context of a single healthcare organization. While existing studies have examined the influence of “asthma severity” or “clinic no-shows” on healthcare utilization for childhood asthma at the community (regional, state, or national) level, there are few organizational (hospital or clinic)-based studies that have concurrently examined the influence of individual biologic, demographic, and behavioral risk factors on unscheduled healthcare use for childhood asthma [14,15]. Moreover, while most existing studies have utilized 12-month timeframes to examine unscheduled healthcare use (emergency or inpatient visits) for childhood asthma, this study examines unscheduled healthcare use for childhood asthma over 12-, 18-, and 24-month timeframes [1,2,16,17]. 

By addressing these gaps in the literature, this study contributes to two streams of literature: (1) self-management effectiveness and (2) healthcare utilization for childhood asthma. Meta-reviews of studies on supported self-management of asthma have emphasized that interventions targeting the combination of patient, provider, and organizational factors have the greatest potential to improve health outcomes compared to targeting patients or providers alone [4,5,6,7,8,9]. This study addresses this need by providing a foundation to identify strategies for improving supported self-management and reducing unscheduled healthcare use for childhood asthma at all three levels. 

Moreover, this study examines the relationship between individual risk factors and unscheduled healthcare use for childhood asthma over 12-, 18-, and 24-month timeframes (at an organizational level). This in turn serves the dual purpose of (1) providing a comprehensive understanding of patterns of unscheduled healthcare use among pediatric outpatients with asthma over an extended two-year period and (2) explicating similarities and differences in characteristics of users of unscheduled healthcare across three different timeframes. Since childhood asthma is a chronic condition, both types of information could enable a healthcare organization to develop a comprehensive, strategic plan for addressing the burden of unscheduled healthcare use for childhood asthma. For example, if clinic no-shows are found to be a significant predictor of unscheduled healthcare use among children in the short term (12 and 18 months), then efforts could be made to implement telehealth or mobile van services for asthma outpatient care to reduce clinic no-shows in the short term across all ages and asthma severity levels [18,19,20,21]. Alternatively, if younger children with severe-persistent asthma are significantly more likely to use unscheduled healthcare in the longer term (24 months), then comprehensive efforts could be undertaken to develop a provider–patient partnership to facilitate patient-centered asthma control (including medication management and environmental control) for younger children with severe-persistent asthma [4,5,6,7,8,9,10,11,22]. Such efforts, in turn, could be undertaken alongside efforts to develop hospital (organization)–community collaborations to address social risk factors for clinic no-shows and unscheduled healthcare use (e.g., financial hardship, housing instability, and transportation, issues) to facilitate effective asthma control and optimal healthcare utilization in the long term for these patients and families [23,24,25]. In summary, this study has the potential to inform the development of short- and long-term interventions for improving SME and reducing unscheduled healthcare for childhood asthma at the (1) patient, (2) provider, and (3) organizational levels.

## 2. Methods

This retrospective study was set in the pediatric outpatient clinics (including allergy-immunology, pulmonology, and general pediatric clinics) at AU Health, a tertiary care academic medical center located in Augusta, Georgia, USA. The study was conducted following approval from the Institutional Review Board (IRB) at Augusta University. The population of interest was children (pediatric patients 0–18 years) who visited the outpatient clinics for asthma between 1 January 2019 and 31 December 2020. Children were included in the study if they belonged to any of the following four severity categories for asthma defined by NHLBI guidelines: (1) intermittent asthma, (2) mild-persistent asthma, (3) moderate-persistent asthma, or (4) severe-persistent asthma. Children with the potential for unrelated respiratory disease, including those with cystic fibrosis, congenital cardiac comorbidities, respiratory disease of prematurity, primary immunodeficiency, and neuromuscular disorders, were excluded from the study. The chart review was conducted by two medical students under the supervision of a pediatric critical care resident and an attending physician.

### 2.1. Data Collection: Dependent Variables (DVs)

The dependent variable (or outcome measure) of this study was “unscheduled healthcare use” (including ED visits or hospitalizations) measured over three retrospective timeframes: (1) 12 months, (2) 18 months, and (3) 24 months. In other words, for children who met the eligibility criteria, data on unscheduled healthcare use were collected over three retrospective timeframes. This in turn translated to three dependent variables (DVs) for the study, distinguished by timeframe for unscheduled healthcare use. Details of DV1, DV2, and DV3 are elaborated upon below.

DV1: Unscheduled healthcare use over 12 months (1 = user; 0 = non-user)

- A user_12 is a patient who has had at least one of any the below types of healthcare encounters over past 12 months:
  ○ Emergency department visit for asthma;
  ○ Inpatient admission for asthma;
  ○ Pediatric Intensive Care Unit (PICU) admission for asthma.
- A non-user_12 is a patient who had none (zero) of all the above types of encounters over past 12 months.

DV2: Unscheduled healthcare use over 18 months (1 = user; 0 = non-user)

- A user_18 is a patient who has had at least one of any the below types of healthcare encounters over past 18 months:
  ○ Emergency department visit for asthma;
  ○ Inpatient admission for asthma;
  ○ Pediatric Intensive Care Unit (PICU) admission for asthma.
- A non-user_18 is a patient who had none (zero) of all the above types of encounters over past 18 months.

DV3: Unscheduled healthcare use over 24 months (1 = user; 0 = non-user)

- A user_24 is a patient who has had at least one of any the below types of healthcare encounters over past 24 months:
  ○ Emergency department visit for asthma;
  ○ Inpatient admission for asthma;
  ○ Pediatric Intensive Care Unit (PICU) admission for asthma.
- A non-user_24 is a patient who had none (zero) of all the above types of encounters over past 24 months.

### 2.2. Data Collection: Independent Variables (IVs)

The following data on individual risk factors (independent variables) were collected for eligible children (users and non-users) of unscheduled healthcare in each of the three retrospective timeframes. It would be relevant to note that data collection timeframes for clinic no-shows and clinic appointment cancellations corresponded to the data collection timeframes for unscheduled healthcare use. For example, for DV1 (unscheduled healthcare use over a 12-month retrospective period), data on clinic no-shows and clinic appointment cancellations were also collected over a 12-month retrospective period.

1. Individual demographic characteristics, including age 0–<8 years, 8–<13 years, 13–<17 years, and ≥17 years); gender (male or female); race (Caucasian, African American, Hispanic, other); and insurance (Medicaid, private, other).
2. Individual risk factors, including disease (asthma) severity (intermittent, mild-persistent, moderate-persistent, or severe-persistent); BMI, defined as normal (<85%), overweight (85–95%), or obese (>95%); medication adherence (yes or no); smoking (yes or no); 12-month, 18-month, and 24-month clinic no-shows (zero no-shows, 1–2 no-shows, 3–4 no-shows, >4 no-shows); 12-month and 18-month clinic appointment cancellations (zero cancellations, 1–5 cancellations, ≥6 cancellations); and 24-month clinic appointment cancellations (0–<6 cancellations, 6–10 cancellations, ≥11 cancellations). It would be relevant to note that medication adherence was defined as a yes (1) or no (0) binary variable. This was the best way to accomplish this for our study, which relied entirely on medical record documentation. For our study sample, the documentation related to medication adherence was complete to the point of being able to distinguish between whether medication adherence was present to any degree (1) or not present at all (0) for all study subjects. Beyond this, the documentation was not available to qualitatively categorize various additional levels of medication adherence (e.g., from very low to very high) reliably and consistently for all study subjects. Correspondingly, a binary definition was determined to be most appropriate for the purpose of this study.

### 2.3. Data Analysis

Differences between users and non-users of unscheduled healthcare (based on in-dividual risk factors) were assessed using multiple logistic regression (MLR) analysis [26] for each of the three dependent variables (DV1, DV2, and DV3) using JMP Pro 16 Software. Essentially, in MLR analysis, we try to find out if the independent variables will collectively predict the logarithmic odds of users and non-users (called the logit of the DV). The MLR analysis results in turn included the following components for each DV: (1) whole model test provides an assessment of whether the MLR model is a good fit to the data. A large Chi-squared test statistic (or a small *p*-value) indicates that the MLR model overall fits the data well. (2) Parameter estimates provide the estimate of each coefficient of the independent variables in the MLR model. For polychotomous independent variables, the results include the coefficient estimate of each level (to the reference level) of the independent variable. When the parameter estimate is found to be significant, it implies that the corresponding design variable is significantly helping predict the logit of the dependent variable, while other independent variables are in the model. (3) Effect likelihood ratio tests give the effect of each dichotomous or polychotomous independent variable as a whole variable. When the effect is significant, indicated by a large Chi-squared estimate to the likelihood ratio test or a small *p*-value for the Chi-squared test statistics, the corresponding independent variable as a whole is significantly contributing to the MLR model, given other variables in the model. (4) Odds ratios for independent variables, which gives the odds ratio of users to non-users for the independent variable in discussion. These four components of the MLR analysis output provide a comprehensive assessment of how the model fits the data and how each independent variable contributes to the overall logit of users to non-users. 

It would be relevant to note that the results of contingency table analysis on differences between users and non-users of unscheduled healthcare (based on individual risk factors) for DV1, DV2, and DV3 are included in the Supplementary Materials. Table S1 summarizes the results of contingency table analysis for DV1, Table S2 for DV2, and Table S3 for DV3. Tables S1–S3 in turn are intended to serve as a supplement to the results of multiple logistic regression analysis for the three dependent variables (DV1, DV2, and DV3, respectively).

A receiver operating characteristic (ROC) curve was also generated for each model to depict the performance of the logistic regression model. An area under curve (AUC) of greater than 50% indicates that the model is effective in differentiating between the two groups being analyzed (in this case, users and non-users of unscheduled healthcare). The closer the AUC is to 100%, the stronger the performance of the model. 

**Figure 1:**
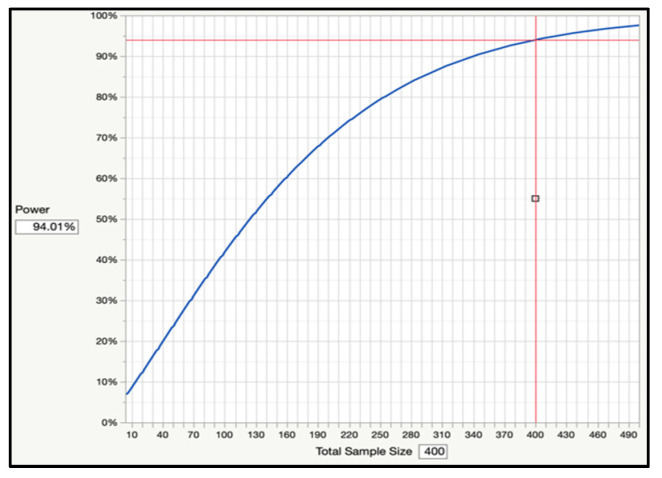
Power Analysis for Sample Size Adequacy.

At the start of the study, a power analysis for sample size adequacy was performed based on proportions of users and non-users of unscheduled healthcare for childhood asthma over 18 months, i.e., 131 users (32%) and 279 non-users (68%) in a total sample of 410 unique pediatric outpatients. The power analysis revealed that a sample size of 400 or higher would yield at least 94.52% power to detect a difference of 0.16 or greater between users and non-users, based on a significance level of 0.05 (SAS 9.4) (See Figure 1).

## 3. Results

Between 1 January 2019 and 31 December 2020, a total of 410 unique children visited AU Health pediatric outpatient clinics for asthma. Of these, the breakdown of users and non-users of unscheduled healthcare for childhood asthma is outlined below for the three dependent variables, DV1, DV2, and DV3:

- A total of 105 children were users of unscheduled healthcare over the previous 12 months, while 305 children were non-users over the previous 12 months. In other words, 26% of the children either visited the ED or were hospitalized for asthma at least once over the previous 12 months, while 74% of the children had zero unscheduled healthcare encounters (ED or inpatient) over the previous 12 months.
- A total of 131 children were users of unscheduled healthcare over the previous 18 months, while 279 children were non-users over the previous 18 months. In other words, 32% of the children either visited the ED or were hospitalized for asthma at least once over the previous 18 months, while 68% of the children had zero unscheduled healthcare encounters (ED or inpatient) over the previous 18 months.
- A total of 147 children were users of unscheduled healthcare over the previous 24 months, while 263 children were non-users over the previous 24 months. In other words, 36% of the children either visited the ED or were hospitalized for asthma at least once over the previous 24 months, while 64% of the children had zero unscheduled healthcare encounters (ED or inpatient) over the previous 24 months.

### 3.1. Summary Characteristics of Study Population

Table 1 provides the summary data on all dependent and independent variables of interest to this study.

### 3.2. Results for Dependent Variable 1 (DV1)

The two tables below summarize the results of logistic regression analysis for DV1. Table 2A summarizes Part 1 of the logistic regression output, including the whole model test, parameter estimates, and effect likelihood ratio, while Table 2B summarizes Part 2 of the logistic regression output, i.e., odds ratios for each independent variable. For the logistic regression parameter estimates (Table 2A), the reference values for the independent variables (indicated in parentheses) were as follows: asthma severity (1 = “intermittent asthma”); age (1 = “0–<8 years”); race (W = “White”); gender (M = “male”); BMI (<85% = “normal”); insurance (P = “private”); medication adherence (0 = none or absent); smoking (N = no smoking or exposure to smoking); clinic no-shows over the previous 12 months (1 = “zero clinic no-shows”); and cancelled appointments over the previous 12 months (1 = ”zero cancelled appointments”).

Taking into consideration all components of the logistic regression output for DV1 (summarized in Table 2A,B), three independent variables emerged as statistically significant predictors of unscheduled healthcare use over the previous 12 months: (1) asthma severity, (2) clinic no-shows over the previous 12 months, and (3) clinic cancelled appointments over the previous 12 months. 

With respect to asthma severity, as indicated in the parameter estimates (Table 2A), children with severe-persistent asthma were significantly more likely to be users of unscheduled healthcare over the previous 12 months compared to children with intermittent asthma. Likewise, children with moderate-persistent asthma were significantly more likely to be users of unscheduled healthcare over the previous 12 months compared to children with intermittent asthma. Asthma severity also emerged as statistically significant predictor of unscheduled healthcare use over the previous 12 months in the effect likelihood ratio tests. Echoing these results, the odds ratios for asthma severity (Table 2B) shows that children with severe-persistent asthma (Category 4) had significantly higher odds of using unscheduled healthcare compared to children with intermittent asthma (Category 1) and children with mild-persistent asthma (Category 2). Similarly, children with moderate-persistent asthma (Category 3) had significantly higher odds of using unscheduled healthcare compared to children with intermittent asthma (Category 1) and children with mild-persistent asthma (Category 2).

Both clinic no-shows over the previous 12 months and clinic cancelled appointments over the previous 12 months emerged as statistically significant predictors of unscheduled healthcare use over the previous 12 months in the effect likelihood ratio tests. Supplementing these results, the odds ratios for clinic cancelled appointments over the previous 12 months revealed that children in Category 2 (1–5 cancelled appointments) were significantly more likely to use unscheduled healthcare for asthma over the previous 12 months compared to children in Category 1 (zero cancelled appointments).

### 3.3. Results for Dependent Variable 2 (DV2)

The two tables below summarize the results of logistic regression analysis for DV2. Table 3A summarizes Part 1 of the logistic regression output, including the whole model test, parameter estimates, and effect likelihood ratio, while Table 3B summarizes Part 2 of the logistic regression output, i.e., odds ratios for each independent variable. For the logistic regression parameter estimates, the reference values for the independent variables (indicated in parentheses) were as follows: asthma severity (1 = “intermittent asthma”); age (1 = “0–<8 years”); race (W = “White); gender (M = “male); BMI (<85% = “normal”); insurance (P = “private”); medication adherence (0 = none or absent); smoking (N = no smoking or exposure); clinic no-shows over the previous 18 months (1 = “zero clinic no-shows”); cancelled appointments over the previous 18 months (1 = ”zero cancelled appointments”).

Taking into consideration all components of the logistic regression output for DV2 (summarized in Table 3A,B), three independent variables emerged as statistically significant predictors of unscheduled healthcare use over the previous 18 months: (1) asthma severity, (2) age, and (3) clinic no-shows over the previous 18 months. 

With respect to asthma severity, as indicated in the parameter estimates (Table 3A), children with severe-persistent asthma were significantly more likely to be users of unscheduled healthcare over the previous 18 months compared to children with intermittent asthma. Likewise, children with moderate-persistent asthma were significantly more likely to be users of unscheduled healthcare over the previous 18 months compared to children with intermittent asthma. Asthma severity also emerged as a statistically significant predictor of unscheduled healthcare use over the previous 18 months in the effect likelihood ratio tests. Echoing these results, the odds ratios for asthma severity (Table 3B) shows that children with severe-persistent asthma (Category 4) had significantly higher odds of using unscheduled healthcare compared to children with intermittent asthma (Category 1) and children with mild-persistent asthma (Category 2). Similarly, children with moderate-persistent asthma (Category 3), had significantly higher odds of using unscheduled healthcare compared to children with intermittent asthma (Category 1) and children with mild-persistent Asthma (Category 2).

Age of child and clinic no-shows over the previous 18 months also emerged as statistically significant predictors of unscheduled healthcare use in the effect likelihood ratio tests. Supplementing these results, the odds ratios for age revealed that older children in the 13–<17 years age group (Category 3) were significantly less likely to use unscheduled healthcare over the previous 18 months compared to younger children in the 0–<8 years age group (Category 1). With respect to clinic no-shows over the previous 18 months, the odds ratios revealed that children in Category 3 (3–4 clinic no-shows) were significantly more likely to use unscheduled healthcare for asthma over the previous 18 months compared to children in Category 1 (zero clinic no-shows).

### 3.4. Results for Dependent Variable 3 (DV3)

The two tables below summarize the results of logistic regression analysis for DV3. Table 4A summarizes Part 1 of the logistic regression output, including the whole model test, parameter estimates, and effect likelihood ratio, while Table 4B summarizes Part 2 of the logistic regression output, i.e., odds ratios for each independent variable. For the logistic regression parameter estimates, the reference values for the independent variables (indicated in parentheses) were as follows: asthma severity (1 = “intermittent asthma”); age (1 = “0–<8 years”); race (W = “White”); gender (M = “male”); BMI (<85% = “normal”); insurance (P = “private”); medication adherence (0 = none or absent); smoking (N = no smoking or exposure); clinic no-shows over the previous 24 months (1 = “zero clinic no-shows”); cancelled appointments over the previous 24 months (1 = ”zero cancelled appointments”).

Taking into consideration all components of the logistic regression output for DV3 (summarized in Table 4A,B), two independent variables emerged as statistically significant predictors of unscheduled healthcare use over the previous 24 months: (1) asthma severity and (2) age of child. 

With respect to asthma severity, as indicated in the parameter estimates (Table 4A), children with severe-persistent asthma were significantly more likely to be users of unscheduled healthcare over the previous 24 months compared to children with intermittent asthma. Likewise, children with moderate-persistent asthma were significantly more likely to be users of unscheduled healthcare over the previous 24 months compared to children with intermittent asthma. Asthma severity also emerged as a statistically significant predictor of unscheduled healthcare use over the previous 24 months in the effect likelihood ratio tests. Echoing these results, the odds ratios for asthma severity (Table 4B) show that children with severe-persistent asthma (Category 4) had significantly higher odds of using unscheduled healthcare compared to children with intermittent asthma (Category 1), children with mild-persistent asthma (Category 2), and children with moderate-persistent asthma (Category 3). Similarly, children with moderate-persistent asthma (Category 3) had significantly higher odds of using unscheduled healthcare compared to children with intermittent asthma (Category 1) and children with mild-persistent asthma (Category 2).

With respect to age of child, as indicated in the parameter estimates (Table 4A), older children in the 13–<17 years age group (Category 3) were significantly less likely to use unscheduled healthcare over the previous 24 months compared to younger children in the 0–<8 years age group (Category 1). Age of child also emerged as a statistically significant predictor of unscheduled healthcare use in the effect likelihood ratio tests. Supplementing these results, the odds ratios for age revealed that older children in both the ≥17 years age group (Category 4) and children in the 13–<17 years age group (Category 3) were significantly less likely to use unscheduled healthcare over the previous 24 months compared to younger children in the 0–<8 years age group (Category 1). Children in the 13–<17 years age group (Category 3) were also significantly less likely to use unscheduled healthcare over the previous 24 months compared to younger children in the 8–<13 years age group (Category 2).

### 3.5. Overall Significance of Results for DV1, DV2, and DV3

**Figure 2:**
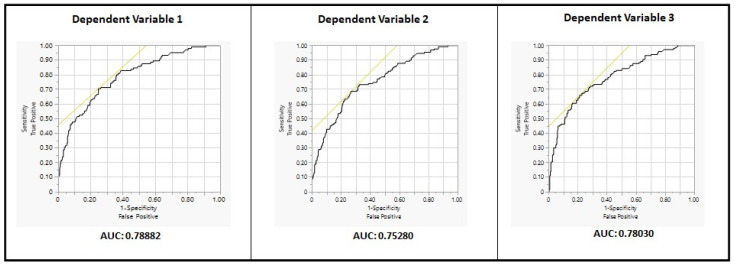
ROC curves for all three logistic regression models (DV1, DV2, and DV3).

As shown in Table 2A, Table 3A and Table 4A, the whole model test for logistic regression was significant for all three dependent variables, indicating that each logistic regression model provides a better fit to the data than a model that contains no independent variables. In addition, Figure 2 depicts the receiver operating characteristic (ROC) curve for all three logistic regression models (DV1, DV2, and DV3). As shown in Figure 2, the area under curve (AUC) is 75% or higher for all three models, indicating that all three models are effective (strong) in differentiating between the two groups being analyzed, i.e., users, and non-users of unscheduled healthcare (over the previous 12 months, 18 months, and 24 months). As indicated earlier, results from contingency table analysis for DV1, DV2, and DV3 are included in the supplementary materials (in Tables S1–S3, respectively). These tables are intended to serve as a supplement to results from logistic regression for DV1, DV2, and DV3, respectively.

## 4. Discussion

To summarize the results, Table 1 shows that most children in the study population were younger (with 50% under 8 years of age), male, African American, and on Medicaid insurance. Most were in the lower asthma severity categories (intermittent or mild-persistent). In addition, with respect to the breakdown of users and non-users by timeframe, the data show that while DV2 captured 25% more users of unscheduled healthcare over DV1 (i.e., 131 over 105), DV3 only captured 12% more users than DV2. Correspondingly, while DV3 could be helpful in planning and developing strategies for long-term asthma management, DV2 may be an ideal indicator (among the three DVs) of unscheduled healthcare use for childhood asthma, particularly with respect to use and re-use of emergency and inpatient healthcare services for childhood asthma. It would also be more consistent with the existing literature on asthma healthcare use, which has mostly relied on 12- or 18-month periods for assessing unscheduled healthcare use for childhood asthma [1,2,16,17].

Moving on to the results of logistic regression analysis, asthma severity emerged as a significant positive predictor of unscheduled healthcare use across all three timeframes. Moreover, the results indicated a linear effect in that, children with severe-persistent asthma had significantly higher odds of using unscheduled healthcare compared to children with intermittent asthma, mild-persistent asthma, and moderate-persistent asthma. Similarly, children with moderate-persistent asthma had significantly higher odds of using unscheduled healthcare compared to children with intermittent asthma and mild-persistent asthma. It would be relevant to note, however, that results from contingency table analysis for all three DVs (included in the Supplementary Materials) indicated that there were users and non-users of unscheduled healthcare in all four asthma severity categories across all timeframes (DVs). For example, as shown in Table S2 for DV2 (18-month retrospective timeframe), there were 14 users and 8 non-users of unscheduled healthcare in the severe-persistent asthma category; 60 users and 58 non-users in the moderate-persistent asthma category; and 9 users and 49 non-users in the intermittent asthma category. This suggests that while asthma severity is clearly a driver of unscheduled healthcare use, it only provides a partial explanation of unscheduled healthcare use, since several children in each asthma severity category, including severe-persistent and moderate-persistent asthma categories managed to refrain from using unscheduled healthcare over the previous 12, 18, and even 24 months.

Like asthma severity, clinic no-shows and clinic cancellations emerged as significant positive predictors of unscheduled healthcare use for childhood asthma in the shorter (12- and 18-month timeframes). Children with 1–5 cancelled appointments were significantly more likely to use unscheduled healthcare for asthma over the previous 12 months compared to children zero cancelled appointments. Likewise, children with 3–4 clinic no-shows were significantly more likely to use unscheduled healthcare for asthma over the previous 18 months compared to children with zero clinic no-shows.

In contrast, age of child emerged as a significant negative predictor, with older children significantly less likely to use unscheduled healthcare for childhood asthma compared to younger children in the longer terms, 18 and 24 months. For example, older children in both the ≥17 years age group and 13–<17 years age group were significantly less likely to use unscheduled healthcare over the previous 24 months compared to the youngest children in the 0–<8 years age group. Similarly, children in the 13–<17 years age group were significantly less likely to use unscheduled healthcare over the previous 24 months compared to children in the 8–<13 years age group.

Notably, the whole model test for logistic regression was significant for all three dependent variables. This indicates that each logistic regression model provided a better fit to the data compared to a model containing no independent variables.

### 4.1. Influence of Social Determinants of Health on Clinic No-Shows for Childhood Asthma

The attention to clinic no-shows as an individual (behavioral) risk factor in this study serves a dual purpose in providing a window to understanding the role of social determinants of health in impacting clinic no-shows and, thereby, unscheduled healthcare use for childhood asthma. 

In recent years, considerable attention has been paid to the association between social determinants of health and missed well-child visits for chronic diseases [23,24,25]. For asthma and other chronic diseases, children receive comprehensive assessments of their physical and behavioral health wellbeing during outpatient clinic (well) visits, and these visits are often used by healthcare providers to identify unmet healthcare needs, guide parents, and initiate early treatment or referral to mitigate adverse health outcomes. Complementing the results of this study, other studies have linked clinic or well-child visits to a lower likelihood of emergency visits and hospitalizations for chronic diseases, including asthma [27,28].

In addition to monitoring child health and wellness, clinic visits could be used to identify the influence of adverse social determinants of health on child health outcomes. Adverse social determinants such as socio-economic and financial hardship have been linked to missed well-child visits and poor outcomes, including childhood asthma and childhood obesity [29,30,31]. In addition, low caregiver support has been linked to worse child health outcomes, including childhood asthma [32,33].

For example, one study found that financial hardship, housing instability, caregiver’s educational attainment of high school or less, and no/poor childcare were associated with missed well-child visits for asthma. In multi-variate regression analysis, having Medicaid insurance and unstable housing were both associated with missed well-child visits. Likewise, a greater percentage of patients with caregivers who reported low social support had missed visits compared to those with high social support. However, when social support was added to the logistic regression model, both Medicaid and unstable housing were no longer associated with missed well-child visits [24]. This suggests that healthcare providers and organizations could greatly benefit from developing a comprehensive understanding of the relationship between adverse social determinants and clinic no-shows for childhood asthma to identify effective long-term strategies for asthma control and optimal healthcare use for childhood asthma at an organizational level.

### 4.2. Implications for Practice

The results provide a foundation for developing short- and long-term strategies for improving supported self-management and reducing unscheduled healthcare use of childhood asthma at patient, provider, and organizational levels. For example, the study found that clinic no-shows and clinic cancellations for regular asthma outpatient care were significant positive predictors of unscheduled healthcare use for childhood asthma in the short term, 12 and 18 months. Results of contingency table analysis (included in the Supplementary Materials) also showed that there were users and non-users of unscheduled healthcare across all levels of asthma severity in the short term (12 and 18 months) and the long term (24 months). 

This suggests that efforts to reduce clinic no-shows among children across all levels of asthma severity (e.g., by implementing telehealth or mobile van services for asthma outpatient care and well visits) could be an effective strategy for reducing unscheduled healthcare use in the short term at an organizational level. Introducing telehealth and mobile van options for asthma outpatient care may be especially relevant for reducing unscheduled health care use among children with lower and moderate levels of asthma severity in the short term. For example, to reduce no-show rates for childhood asthma care, one clinic serving low-income communities in Chicago, USA, introduced a telehealth option for off-site parents to attend their child’s on-site appointment. Following the introduction of the telehealth options, no-show rates decreased significantly from 36% to 7.9%–18% per month over a 10-month implementation period. Post-telehealth surveys completed by parents revealed this version of telehealth improved access to care for their child, saved them time, and was simple to use [34].

In addition to implementing telehealth services, a growing number of healthcare providers have initiated screening for social determinants of health, given the positive impact this could have on overall health status [35,36]. Recent research has also demonstrated that patients and families participating in such programs have reported reduced unmet social need and improved child health outcomes [37].

Results of existing research discussed in the earlier sub-section suggest that caregiver social support may also be an important avenue for interventions geared toward improving clinic (well-child) visits for low-income families experiencing adverse social determinants of health. Adequate caregiver social support has the potential to improve clinic visits by helping to reduce caregiver depression [38].

In summary, social determinants of health could negatively impact clinic (well) visits for childhood asthma outpatient care, highlighting the importance of screening for social determinants to improve outpatient care utilization and reduce health disparities in childhood asthma. In addition to screening and referral to services that address financial hardship, research suggests that screening for social support may be a potential mechanism to further identify patients at risk for unscheduled healthcare use for childhood asthma.

Lastly, results indicate that younger children with higher asthma severity are significant more likely to use unscheduled healthcare in the long term (24 months). This in turn suggests that healthcare organizations may benefit most from implementing a comprehensive strategy spanning interventions across patient, provider, and organizational levels for reducing unscheduled healthcare use among younger children with higher asthma severity in the long term. 

Key components of such a comprehensive strategy would include: (1) patient-centered asthma education strategies, e.g., teach-back techniques targeted toward patients at risk for clinic no-shows and unscheduled health care use; (2) provider education related to the benefits of partnering with families of younger children with severe-persistent asthma to facilitate patient-centered asthma control (including medication management and environmental control) in the long term; and (3) organizational initiatives to (i) implement telehealth and/or mobile van services for asthma outpatient care in the short term and (ii) undertake hospital–community collaborations to address SDoH associated with unscheduled healthcare use to facilitate effective asthma control and optimal healthcare use in the long term. For example, in addition to providing comprehensive self-management education to asthma patients and families in the clinic setting, healthcare providers and organizations could undertake proactive community-based interventions to target SDoH impacting childhood asthma, e.g., school involvement, by reaching out to educate children at school, where they spend most of their time, and by offering behavior education counseling for school personnel to fill the access to care gap. Schools could also be educated on the benefits of adopting asthma-friendly policies to empower children to adhere to their asthma-action plans, such as stocking quick-relief medications, allowing older children to carry controller and rescue medicines, and allowing children to exercise indoors when air quality is poor. Asthma healthcare providers could also work to establish collaborations with community-based pharmacies and community health workers to ensure that children with asthma receive the necessary services. Healthcare providers and organizations could also play a proactive role in advocating for policy reform related to childhood asthma, including provider reimbursement reform to ensure payment for asthma self-management education and other efforts undertaken by providers to improve access to asthma medications and asthma control services in the community. In summary, multi-sectoral engagement, including collaboration across hospitals, primary care physicians, pharmacies, schools, community health workers, governmental and public health organizations, and patients and families may often be necessary for ensuring effective management of childhood asthma and optimal healthcare use in the long term [39,40].

### 4.3. Implications for Future Research

This study contributes to the literature on asthma healthcare utilization by examining the concurrent impact of ten individual level risk factors on unscheduled healthcare use for childhood asthma at an organizational level. In doing so, it helps to highlight the statistically significant influence of asthma severity, age of child, and clinic no-shows on unscheduled healthcare use for childhood asthma over three timeframes (12, 18, and 24 months). The attention to three timeframes also helps to identify short- and long-term strategies for reducing unscheduled healthcare use for childhood asthma at three (patient, provider, and organizational) levels. This in turn helps to address a need identified in the literature for a comprehensive set of interventions spanning all three levels for improving supported self-management, reducing unscheduled healthcare use, and improving health outcomes for childhood asthma [4,5,6,7,8,9].

From a future research standpoint, it would be helpful to expand upon the base of risk factors (independent variables) examined to include multiple determinants of asthma SME informed by the “holistic framework” for assessing asthma SME and unscheduled healthcare use (discussed earlier). While clinic no-shows can provide a window to understanding the social risk factors impacting unscheduled healthcare use, primary data collection on risk factors at multiple levels, including socio-economic status, housing instability, and financial hardship, can help to inform a comprehensive framework for tackling the challenge of unscheduled healthcare use for childhood asthma. Future research is also needed to identify interventions to increase caregiver social support and its effect on unscheduled healthcare use for childhood asthma. Another avenue for future research would be the translation of research to practice, i.e., implementation of strategies and interventions identified in this (and similar studies) into practice, coupled with clinical trials to assess the effectiveness of these interventions in improving SME and reducing the burden of unscheduled healthcare use for childhood asthma.

### 4.4. Study Limitations

This study addresses a gap in the literature by concurrently examining the influence of a comprehensive set of individual biologic, demographic, and behavioral risk factors on unscheduled healthcare use for childhood asthma at an organizational level over three different retrospective timeframes (12, 18, and 24 months). While existing studies have examined the influence of individual risk factors such as asthma severity or clinic no-shows on healthcare use at a community level, there are few studies of this nature that have been conducted at an organizational (hospital/clinic) level [14,15]. Meta-reviews of studies on supported self-management of asthma have emphasized that interventions targeting the combination of patient, provider, and organizational factors have the greatest potential to improve health outcomes, compared to targeting patients or providers alone [4,5,6,7,8,9,10,11]. By virtue of its scope and design, this study directly addresses this need by providing a foundation for generating short- and long-term strategies for improving supported self-management and reducing unscheduled healthcare use for childhood asthma at patient, provider, and organizational levels.

However, it would be important to also acknowledge study limitations. To begin with, this study relies on a retrospective review of medical records, which restricts data collected to that available in the medical record. Despite being guided by a “holistic framework” for assessing factors influencing SME and unscheduled healthcare use for childhood asthma (which would ideally span multiple levels of data collection, including individual, social, community, organizational, community, and environmental levels), this study is restricted to data collection on individual risk factors. Despite this limitation, the study was able to leverage data collected on select individual behavioral risk factors such as clinic no-shows and clinic cancellations, which helped serve a dual purpose in providing a window to understanding the influence of SDoH on SME and unscheduled healthcare use for childhood asthma (as discussed in Section 4.1). Additionally, despite relying on a retrospective review of medical records, this study benefitted from a robust sample size. As discussed in the sub-section on power analysis under methodology, the sample size of 410 patients was sufficient to yield at least 94.52% power to detect a difference of 0.16 or greater between users and non-users of unscheduled healthcare for childhood asthma based on a significance level of 0.05. 

Another methodological limitation that needs to be acknowledged is that this study may not have captured patient visits to other emergency departments or urgent care centers in the community. However, this concern is mitigated by the fact that the study institution is the second largest children’s hospital in the state of Georgia and that past studies at this institution have established that over 95% of the pediatric outpatient population relies on the same health system (academic medical center) for primary, secondary, and tertiary care [12,13]. This concern is also mitigated by the fact that most of the study population was on Medicaid and that past studies have shown that patients on Medicaid are less prone to seek care from a wider network of providers due to insurance limitations [41]. 

A key strength of the study was the reliance on clinical experts for retrospective chart review (including two medical students, a critical care resident, and an attending supervising physician). The study also benefited from an adequately powered sample size for the logistic regression analysis. The study location may also be viewed as a strength. In 2015, the Asthma and Allergy Foundation of America recognized Augusta, Georgia, as one of the “Top Ten Asthma Capitals of the US” [42]. Augusta is known to have elevated rates of morbidity and mortality from a variety of chronic diseases, especially asthma. Correspondingly, our sample of asthma-vulnerable children may be highly representative of other high-risk rural or inner-city US outpatient settings for asthma treatment, and the higher asthma severity of the patient base served by the medical center may be directly relevant to understanding the problem of unscheduled healthcare use for childhood asthma, as echoed by the Pareto Principle on healthcare use: 80% of unscheduled (costly) healthcare use could be attributed to the 20% severely ill populace [43].

## 5. Conclusions

This study examines the influence of various individual demographic and risk factors on the use of unscheduled healthcare (including emergency and inpatient visits) among children with asthma over three retrospective timeframes (12, 18, and 24 months), at an academic health center. While existing studies have examined the influence of individual risk factors such as asthma severity or clinic no-shows on healthcare use at a community level, there are few studies of this nature that have been conducted at an organizational (hospital/clinic) level. Logistic regression analysis of the effect of individual risk factors, revealed that asthma severity, age of child, and clinic no-shows were statistically significant predictors of unscheduled healthcare use for childhood asthma. The results provide a foundation for identifying short- and long-term strategies for improving supported self-management and reducing unscheduled healthcare use for childhood asthma at the patient, provider, and organizational levels, e.g., implementing telehealth services for asthma outpatient care to reduce clinic no-shows across all levels of asthma severity in the short term; developing a provider–patient partnership to enable patient-centered long-term asthma control; and identifying hospital–community linkages to address social risk factors influencing clinic no-shows and unscheduled healthcare use among younger children with higher asthma severity in the long term. A fruitful avenue for future research would be the translation of research to practice, i.e., the implementation of strategies and interventions identified in this study into practice, coupled with clinical trials, to assess the effectiveness of these interventions in reducing the burden of unscheduled healthcare use for childhood asthma.

## Figures and Tables

**Table 1:** Summary data on study variables.

Parameter	N	%
DV 1 (12 Months)		
User	105	26%
Non-User	305	74%
Total	410	100%
DV 2 (18 Months)		
User	131	32%
Non-User	279	68%
Total	410	100%
DV 3 (24 Months)		
User	147	36%
Non-User	263	64%
Total	410	100%
Individual Demographics (IVs)		
Age: 0–<8 years	205	50%
Age: 8–<13 years	141	34%
Age: 13–<17 years	47	11%
Age: ≥17 years	17	4%
Total	410	100%
Gender: Male	271	66%
Gender: Female	139	34%
Total	410	100%
Race: Caucasian	135	33%
Race: African American	223	54%
Race: Hispanic	21	5%
Race: Other	31	8%
Total	410	100%
Insurance: Medicaid	231	56%
Insurance: Private	175	43%
Insurance: Other	4	1%
Total	410	100%
Individual Risk Factors (IVs)		
Asthma Severity: Intermittent	58	14%
Asthma Severity: Mild-Persistent	212	52%
Asthma Severity: Moderate-Persistent	118	29%
Asthma Severity: Severe-Persistent	22	5%
Total	410	100%
BMI: Normal	239	58%
BMI: Overweight	59	14%
BMI: Obese	112	27%
Total	410	100%
Medication Adherence: Yes	320	78%
Medication Adherence: No	90	22%
Total	410	100%
Smoking: Yes	72	18%
Smoking: No	338	82%
Total	410	100%
Clinic No-Shows: 0 (Zero)	341	83%
Clinic No-Shows: 1–2	53	13%
Clinic No-Shows: 3–4	8	2%
Clinic No-Shows: >4	8	2%
Total	410	100%
Clinic Cancellations: 0 (Zero)	218	53%
Clinic Cancellations: 1–5	182	44%
Clinic Cancellations: ≥6	10	2%
Total	410	100%

**Table 2:** (**A**) Logistic Regression Output for DV1 (Part 1). (**B**) Logistic Regression Output for DV1: Odds of User vs. Non-User (Part 2).

**(A)**						
**Whole Model Test**						
**Model**	**Log Likelihood**	**DF**	**Chi Square**		**Prob > Chi Sq**	
**Difference**	47.73091	21	95.46182		<0.0001 *	
Full	185.5326					
Reduced	233.26351					
**Parameter Estimates**						
**Term**	**Estimate**	**Std Error**	**Chi Square**	**Prob > Chi Sq**	**Lower 95%**	**Upper 95%**
Intercept	3.10925547	286.18073	0	0.9913	−1.337641	564.01317
Asthma Severity (Severe-Persistent)	1.00766313	0.4202947	5.75	0.0165 *	0.1822147	1.8481486
Asthma Severity (Moderate-Persistent)	0.73348999	0.2345889	9.78	0.0018 *	0.2761057	1.1990824
Asthma Severity (Mild-Persistent)	−0.8439822	0.2479135	11.59	0.0007 *	−1.335839	−0.360113
Age (17 years or more)	−0.3849129	0.5031052	0.59	0.4442	−1.458727	0.5515176
Age (13 to <17 years)	−0.425855	0.3792272	1.26	0.2615	−1.207428	0.2944103
Age (8 to <13 years)	0.26933076	0.2647422	1.03	0.309	−0.242509	0.801635
Gender (Female)	0.19893506	0.1379018	2.08	0.1491	−0.072656	0.4694543
Race (African American)	−0.0912106	0.2567094	0.13	0.7224	−0.590236	0.4222511
Race (Hispanic)	0.25553467	0.472329	0.29	0.5885	−0.730232	1.1475258
Race (Other)	−0.2024715	0.4405372	0.21	0.6458	−1.134801	0.6189104
Insurance (Other)	−0.1254631	0.8980123	0.02	0.8889	−2.291412	1.5013845
Insurance (Medicaid)	0.26627943	0.4706286	0.32	0.5715	−0.594814	1.3755291
BMI (85%–95%)	−0.0760498	0.2736893	0.08	0.7811	−0.634293	0.4452441
BMI (>95%)	−0.2592004	0.2333955	1.23	0.2668	−0.726666	0.1929795
Medication Adherence (Yes)	−0.1082721	0.1593159	0.46	0.4968	−0.416859	0.2100099
Smoking (Yes)	0.06674975	0.1729105	0.15	0.6995	−0.280879	0.400096
12-Month Clinic No-Shows (>4)	12.3212843	858.5404	0	0.9885	1.0119037	1695.0295
12-Month Clinic No-Shows (3–4)	−3.5081953	286.18072	0	0.9902	−564.4121	0.8880258
12-Month Clinic No-Shows (1–2)	−4.2117577	286.18031	0	0.9883	−565.1149	556.69135
12-Month Clinic Cancellations (>6)	−0.135352	0.5865781	0.05	0.8175	−1.379394	0.9998991
12-Month Clinic Cancellations (1–5)	0.49501316	0.3187117	2.41	0.1204	−0.121688	1.1594346
**Effect Likelihood Ratio Tests**						
**Source**	**N parm**	**DF**	**L-R Chi Square**		**Prob > Chi Sq**	
Asthma Severity	3	3	34.3119365		<0.0001 *	
Age	3	3	5.27815849		0.1525	
Gender	1	1	2.06868437		0.1504	
Race	3	3	0.51852276		0.9148	
Insurance	2	2	2.04399283		0.3599	
BMI	2	2	3.75236926		0.1532	
Medication Adherence	1	1	0.45657801		0.4992	
Smoking	1	1	0.14775536		0.7007	
12-Month Clinic No-Shows	3	3	14.0359689		0.0029 *	
12-Month Clinic Cancellations	2	2	9.28535975		0.0096 *	
Notes: For log odds of 1/0 (user/non-user); confidence limits are likelihood-based.						
**(B)**						
**Odds Ratios for Asthma Severity**						
**Level1**	**/Level2**	**Odds Ratio**	**Prob > Chi Sq**		**Lower 95%**	**Upper 95%**
Severe-Persistent	Intermittent	6.7182928	0.0051 *		1.77393	25.443766
Moderate-Persistent	Intermittent	5.1072492	0.0005 *		2.03754	12.801709
Mild-Persistent	Intermittent	1.0546287	0.91		0.4193492	2.6523043
Severe-Persistent	Mild-Persistent	6.370292	0.0015 *		2.0244327	20.045428
Moderate-Persistent	Mild-Persistent	4.8426988	<0.0001 *		2.6266546	8.9283653
Severe-Persistent	Moderate-Persistent	1.3154425	0.6268		0.4356032	3.9723971
**Odds Ratios for Age**						
**Level1**	**/Level2**	**Odds Ratio**	**Prob > Chi Sq**		**Lower 95%**	**Upper 95%**
≥17 years	0–<8 years	0.3959965	0.1752		0.1037825	1.5109793
13–<17 years	0–<8 years	0.3801109	0.0531		0.1426082	1.013156
8–<13 years	0–<8 years	0.7617732	0.3671		0.42171	1.3760603
≥17 years	8–<13 years	0.5198351	0.3412		0.1351193	1.9999255
13–<17 years	8–<13 years	0.4989817	0.1661		0.1865546	1.3346374
≥17 years	13–<17 years	1.0417919	0.9581		0.2258901	4.8046826
**Odds Ratios for Gender**						
**Level1**	**/Level2**	**Odds Ratio**	**Prob > Chi Sq**		**Lower 95%**	**Upper 95%**
Female	Male	1.4886507	0.1491		0.8670186	2.5559785
**Odds Ratios for Race**						
**Level1**	**/Level2**	**Odds Ratio**	**Prob > Chi Sq**		**Lower 95%**	**Upper 95%**
Hispanic	African American	1.4144564	0.5775		0.4175866	4.7910713
Other	African American	0.8947053	0.8481		0.2865822	2.7932566
Other	Hispanic	0.6325436	0.5696		0.1304904	3.0662121
African American	White	0.8786593	0.6788		0.4763782	1.6206494
Hispanic	White	1.2428253	0.7358		0.351526	4.3940263
Other	White	0.7861411	0.6838		0.2469147	2.5029608
**Odds Ratios for Insurance**						
**Level1**	**/Level2**	**Odds Ratio**	**Prob > Chi Sq**		**Lower 95%**	**Upper 95%**
Other	Medicaid	0.6758781	0.7723		0.0475925	9.598397
Other	Private	1.0154718	0.991		0.0712492	14.472914
Medicaid	Private	1.502448	0.1573		0.8546733	2.6411849
**Odds Ratios for BMI**						
**Level1**	**/Level2**	**Odds Ratio**	**Prob > Chi Sq**		**Lower 95%**	**Upper 95%**
Overweight	Normal	0.6627881	0.319		0.2951565	1.4883226
Obese	Normal	0.5518657	0.0718		0.2888932	1.0542155
Obese	Overweight	0.8326427	0.697		0.3311887	2.0933498
**Odds Ratios for Medication Adherence**						
**Level1**	**/Level2**	**Odds Ratio**	**Prob > Chi Sq**		**Lower 95%**	**Upper 95%**
Yes	No	0.805297	0.4968		0.4312571	1.5037509
**Odds Ratios for Smoking**						
**Level1**	**/Level2**	**Odds Ratio**	**Prob > Chi Sq**		**Lower 95%**	**Upper 95%**
No	Yes	0.8750279	0.6995		0.4442818	1.7233967
**Odds Ratios for Clinic No-Shows Over Previous 12 Months**						
**Level1**	**/Level2**	**Odds Ratio**	**Prob > Chi Sq**		**Lower 95%**	**Upper 95%**
>4	0 (Zero)	22356230	0.9882		0	.
3–4	0 (Zero)	2.9836162	0.1908		0.5800966	15.34566
1–2	0 (Zero)	1.4763511	0.2926		0.7146973	3.0497003
>4	1–2	15142896	0.9885		0	.
3–4	1–2	2.0209394	0.4125		0.375618	10.87327
>4	3–4	7492998.2	0.989		0	.
**Odds Ratios for Clinic Cancellations Over Previous 12 Months**						
**Level1**	**/Level2**	**Odds Ratio**	**Prob > Chi Sq**		**Lower 95%**	**Upper 95%**
≥6	0 (Zero)	1.2514578	0.8031		0.2146779	7.2953341
1–5	0 (Zero)	2.3506087	0.0028 *		1.3430675	4.1139863
≥6	1–5	0.5323974	0.4754		0.0942852	3.0062721
* Statistically significant at the alpha = 0.05 level Note: Normal approximations used for ratio confidence limits effects						
Note: Tests and confidence intervals on odds ratios are Wald based						

**Table 3:** (**A**) Logistic Regression Output for DV2 (Part 1). (**B**) Logistic Regression Output for DV2: Odds of User vs. Non-User (Part 2).

**(A)**						
**Whole Model Test**						
**Model**	**Log Likelihood**	**DF**	**Chi Square**		**Prob > Chi Sq**	
**Difference**	41.66268	21	83.32535		<0.0001 *	
Full	215.20282					
Reduced	256.8655					
**Parameter Estimates**						
**Term**	**Estimate**	**Std Error**	**Chi Square**	**Prob > Chi Sq**	**Lower 95%**	**Upper 95%**
Intercept	3.81066446	463.17797	0	0.9934	−0.807645	911.622812
Asthma Severity (Severe-Persistent)	0.88484924	0.4111501	4.63	0.0314 *	0.082496	1.71312545
Asthma Severity (Moderate-Persistent)	0.64232011	0.2240972	8.22	0.0042 *	0.20337465	1.08481296
Asthma Severity (Mild-Persistent)	−0.5811561	0.2252614	6.66	0.0099 *	−1.0265903	−0.1404289
Age (17 years or more)	−0.2516179	0.4551622	0.31	0.5804	−1.2026226	0.61172251
Age (13 to <17 years)	−0.6228092	0.3595594	3	0.0832	−1.3661091	0.05658379
Age (8 to <13 years)	0.30290465	0.243885	1.54	0.2142	−0.1695335	0.79091998
Gender (Female)	0.14081023	0.1261681	1.25	0.2644	−0.1077524	0.38790043
Race (African American)	0.08930488	0.2314633	0.15	0.6996	−0.3610562	0.55047594
Race (Hispanic)	0.09610884	0.4274907	0.05	0.8221	−0.7885718	0.90885011
Race (Other)	−0.0425634	0.3849673	0.01	0.912	−0.8396313	0.68663044
Insurance (Other)	−0.1824744	0.8544395	0.05	0.8309	−2.2945646	1.34496685
Insurance (Medicaid)	0.22120023	0.4450133	0.25	0.6191	−0.5833937	1.29788571
BMI (85%–95%)	0.02552395	0.2432243	0.01	0.9164	−0.4653271	0.49311991
BMI (>95%)	−0.245739	0.2052649	1.43	0.2312	−0.6542757	0.15321765
Medication Adherence (Yes)	−0.1213201	0.1465945	0.68	0.4079	−0.4063745	0.17003355
Smoking (Yes)	0.12812873	0.1568931	0.67	0.4141	−0.1840971	0.43310822
18-Month Clinic No-Shows (>4)	12.659048	1389.533	0	0.9927	−2710.7756	2736.09371
18-Month Clinic No-Shows (3–4)	−3.3340158	463.17793	0	0.9943	−911.14608	904.478051
18-Month Clinic No-Shows (1–2)	−4.529019	463.17777	0	0.9922	−912.34076	903.282722
18-Month Clinic Cancellations (>6)	0.31241103	0.3844156	0.66	0.4164	−0.4447229	1.08317702
18-Month Clinic Cancellations (1–5)	0.07647871	0.2303683	0.11	0.7399	−0.381499	0.5284306
**Effect Likelihood Ratio Tests**						
**Source**	**N parm**	**DF**	**L-R Chi Square**		**Prob > Chi Sq**	
Asthma Severity	3	3	27.6008402		<0.0001 *	
Age	3	3	7.97179927		0.0466 *	
Gender	1	1	1.23964425		0.2655	
Race	3	3	0.6985804		0.8735	
Insurance	2	2	1.09013917		0.5798	
BMI	2	2	2.62511381		0.2691	
Medication Adherence	1	1	0.67794164		0.4103	
Smoking	1	1	0.65971892		0.4167	
18-Month Clinic No-Shows	3	3	12.7120747		0.0053 *	
18-Month Clinic Cancellations	2	2	3.69393572		0.1577	
Notes: For log odds of 1/0 (user/non-user); confidence limits are likelihood-based.						
**(B)**						
**Odds Ratios for Asthma Severity**						
**Level1**	**/Level2**	**Odds Ratio**	**Prob > Chi Sq**		**Lower 95%**	**Upper 95%**
Severe-Persistent	Intermittent	6.2392658	0.0047 *		1.7521237	22.217859
Moderate-Persistent	Intermittent	4.8955831	0.0002 *		2.1155993	11.328579
Mild-Persistent	Intermittent	1.4403083	0.3822		0.6354456	3.2646192
Severe-Persistent	Mild-Persistent	4.3318959	0.0095 *		1.4309161	13.114201
Moderate-Persistent	Mild-Persistent	3.3989827	<0.0001 *		1.947435	5.9324618
Severe-Persistent	Moderate-Persistent	1.2744684	0.6623		0.4291828	3.7845638
**Odds Ratios for Age**						
**Level1**	**/Level2**	**Odds Ratio**	**Prob > Chi Sq**		**Lower 95%**	**Upper 95%**
≥17 years	0–<8 years	0.4390507	0.1829		0.1307521	1.474283
13–<17 years	0–<8 years	0.3029064	0.0123 *		0.1189042	0.7716487
8–<13 years	0–<8 years	0.7644354	0.3243		0.4480939	1.3041048
≥17 years	8–<13 years	0.5743464	0.3728		0.1696332	1.9446298
13–<17 years	8–<13 years	0.3962485	0.0536		0.1548002	1.0142939
≥17 years	13–<17 years	1.4494603	0.6043		0.3560495	5.9006823
**Odds Ratios for Gender**						
**Level1**	**/Level2**	**Odds Ratio**	**Prob > Chi Sq**		**Lower 95%**	**Upper 95%**
Female	Male	1.3252756	0.2644		0.808197	2.1731774
**Odds Ratios for Race**						
**Level1**	**/Level2**	**Odds Ratio**	**Prob > Chi Sq**		**Lower 95%**	**Upper 95%**
Hispanic	African American	1.0068272	0.9904		0.3318184	3.0549875
Other	African American	0.8764564	0.7953		0.3236867	2.3732078
Other	Hispanic	0.8705133	0.8465		0.2137911	3.5445512
African American	White	1.2613155	0.4151		0.7216654	2.2045072
Hispanic	White	1.2699267	0.6832		0.4030005	4.0017658
Other	White	1.1054881	0.8462		0.4014029	3.0445812
**Odds Ratios for Insurance**						
**Level1**	**/Level2**	**Odds Ratio**	**Prob > Chi Sq**		**Lower 95%**	**Upper 95%**
Other	Medicaid	0.6678614	0.7538		0.0535905	8.3230928
Other	Private	0.8661055	0.9112		0.0692616	10.83052
Medicaid	Private	1.2968342	0.3082		0.7866082	2.1380135
**Odds Ratios for BMI**						
**Level1**	**/Level2**	**Odds Ratio**	**Prob > Chi Sq**		**Lower 95%**	**Upper 95%**
Overweight	Normal	0.8230889	0.5984		0.398812	1.6987333
Obese	Normal	0.6275361	0.1112		0.3536754	1.1134547
Obese	Overweight	0.762416	0.5135		0.3379288	1.7201204
**Odds Ratios for Medication Adherence**						
**Level1**	**/Level2**	**Odds Ratio**	**Prob > Chi Sq**		**Lower 95%**	**Upper 95%**
Yes	No	0.7845537	0.4079		0.4416312	1.3937524
**Odds Ratios for Smoking**						
**Level1**	**/Level2**	**Odds Ratio**	**Prob > Chi Sq**		**Lower 95%**	**Upper 95%**
Yes	No	1.2920853	0.4141		0.6985475	2.3899371
**Odds Ratios for Clinic No-Shows Over Previous 18 Months**						
**Level1**	**/Level2**	**Odds Ratio**	**Prob > Chi Sq**		**Lower 95%**	**Upper 95%**
>4	0 (Zero)	38074722	0.9925		0	.
3–4	0 (Zero)	4.3145687	0.0403 *		1.0666393	17.452482
1–2	0 (Zero)	1.3060329	0.4537		0.6495346	2.6260679
>4	1–2	29152958	0.9926		0	.
3–4	1–2	3.3035682	0.109		0.7662447	14.242922
>4	3–4	8824687.7	0.9931		0	.
**Odds Ratios for Clinic Cancellations Over Previous 18 Months**						
**Level1**	**/Level2**	**Odds Ratio**	**Prob > Chi Sq**		**Lower 95%**	**Upper 95%**
≥6	0 (Zero)	2.0163738	0.2387		0.6279514	6.4746466
1–5	0 (Zero)	1.5926009	0.0789		0.9476413	2.6765164
≥6	1–5	1.2660886	0.6883		0.3998737	4.008717
* Statistically significant at the alpha = 0.05 level Note: Normal approximations used for ratio confidence limits effects						
Note: Tests and confidence intervals on odds ratios are Wald based						

**Table 4:** (**A**) Logistic Regression Output for DV3 (Part 1). (**B**) Logistic Regression Output for DV3: Odds of User vs. Non-User (Part 2).

**(A)**						
**Whole Model Test**						
**Model**	**Log Likelihood**	**DF**	**Chi Square**		**Prob > Chi Sq**	
**Difference**	50.60995	21	101.2199		<0.0001 *	
Full	216.94438					
Reduced	267.55433					
**Parameter Estimates**						
**Term**	**Estimate**	**Std Error**	**Chi Square**	**Prob > Chi Sq**	**Lower 95%**	**Upper 95%**
Intercept	−0.5862596	0.5550046	1.12	0.2908	−1.8124974	0.44749185
Asthma Severity (Severe-Persistent)	1.81645034	0.4676356	15.09	0.0001 *	0.9489207	2.80622487
Asthma Severity (Moderate-Persistent)	0.51041062	0.2308243	4.89	0.0270 *	0.05099225	0.96076985
Asthma Severity (Mild-Persistent)	−0.9350318	0.2441205	14.67	0.0001 *	−1.4266689	−0.4649346
Age (17 years or more)	−0.6083886	0.4797115	1.61	0.2047	−1.6013847	0.30829146
Age (13 to <17 years)	−0.6625515	0.345434	3.68	0.0551	−1.3635104	−0.0006684
Age (8 to <13 years)	0.50453293	0.2489767	4.11	0.0427 *	0.02440269	1.00475046
Gender (Female)	0.09898491	0.1267119	0.61	0.4347	−0.1504002	0.34736271
Race (African American)	0.37411119	0.2269068	2.72	0.0992	−0.066627	0.82656267
Race (Hispanic)	−0.1604753	0.4305123	0.14	0.7093	−1.0508162	0.65913693
Race (Other)	−0.0708666	0.377069	0.04	0.8509	−0.8427195	0.64912982
Insurance (Other)	−0.2546501	0.916096	0.08	0.781	−2.4528227	1.36168909
Insurance (Medicaid)	0.30675852	0.4748893	0.42	0.5183	−0.539296	1.4278081
BMI (85%–95%)	0.09283277	0.2364316	0.15	0.6946	−0.3791806	0.55152427
BMI (>95%)	−0.3011504	0.2018629	2.23	0.1357	−0.7028651	0.09082505
Medication Adherence (Yes)	−0.2076972	0.1459336	2.03	0.1547	−0.4939261	0.07973125
Smoking (Yes)	0.25713526	0.1576219	2.66	0.1028	−0.0528441	0.56685475
24-Month Clinic No-Shows (>4)	0.39793737	0.3136794	1.61	0.2046	−0.2138095	1.0228706
24-Month Clinic No-Shows (3–4)	−0.1139315	0.275805	0.17	0.6795	−0.6635257	0.42195555
24-Month Clinic No-Shows (1–2)	0.054728	0.2015985	0.07	0.786	−0.3409418	0.45100075
24-Month Clinic Cancellations (>11)	0.04404362	0.2478175	0.03	0.8589	−0.4471906	0.52808413
24-Month Clinic Cancellations (6–10)	0.25921714	0.1961435	1.75	0.1863	−0.1243559	0.64638514
**Effect Likelihood Ratio Tests**						
**Source**	**N parm**	**DF**	**L-R Chi Square**		**Prob > Chi Sq**	
Asthma Severity	3	3	47.1578898		<0.0001 *	
Age	3	3	13.5841515		0.0035 *	
Gender	1	1	0.60885158		0.4352	
Race	3	3	3.99857532		0.2616	
Insurance	2	2	2.09934652		0.3501	
BMI	2	2	3.15275762		0.2067	
Medication Adherence	1	1	2.01356905		0.1559	
Smoking	1	1	2.64865666		0.1036	
24-Month Clinic No-Shows	3	3	3.4257436		0.3305	
24-Month Clinic Cancellations	2	2	3.79600814		0.1499	
Notes: For log odds of 1/0 (user/non-user); confidence limits are likelihood-based.						
**(B)**						
**Odds Ratios for Asthma Severity**						
**Level1**	**/Level2**	**Odds Ratio**	**Prob > Chi Sq**		**Lower 95%**	**Upper 95%**
Severe-Persistent	Intermittent	24.73649	<0.0001 *		6.0844816	100.56632
Moderate-Persistent	Intermittent	6.7008859	<0.0001 *		2.8733485	15.627019
Mild-Persistent	Intermittent	1.5790087	0.2772		0.6926698	3.5995053
Severe-Persistent	Mild-Persistent	15.665834	<0.0001 *		4.4047056	55.717315
Moderate-Persistent	Mild-Persistent	4.2437295	<0.0001 *		2.4136762	7.461332
Severe-Persistent	Moderate-Persistent	3.6915252	0.0350 *		1.0959504	12.434284
**Odds Ratios for Age**						
**Level1**	**/Level2**	**Odds Ratio**	**Prob > Chi Sq**		**Lower 95%**	**Upper 95%**
≥17 years	0–<8 years	0.2528912	0.0363 *		0.0698011	0.9162321
13–<17 years	0–<8 years	0.2395583	0.0018 *		0.0975783	0.5881244
8–<13 years	0–<8 years	0.7696078	0.3389		0.4499779	1.3162783
≥17 years	8–<13 years	0.3285975	0.091		0.0903994	1.194437
13–<17 years	8–<13 years	0.3112732	0.0112 *		0.126299	0.7671554
≥17 years	13–<17 years	1.0556565	0.9406		0.2541272	4.3852474
**Odds Ratios for Gender**						
**Level1**	**/Level2**	**Odds Ratio**	**Prob > Chi Sq**		**Lower 95%**	**Upper 95%**
Female	Male	1.2189256	0.4347		0.7417585	2.0030504
**Odds Ratios for Race**						
**Level1**	**/Level2**	**Odds Ratio**	**Prob > Chi Sq**		**Lower 95%**	**Upper 95%**
Hispanic	African American	0.5859115	0.3477		0.1919701	1.7882588
Other	African American	0.6408385	0.3669		0.2437878	1.6845549
Other	Hispanic	1.0937463	0.9		0.2704439	4.423397
African American	White	1.6767887	0.0662		0.9659637	2.9106895
Hispanic	White	0.9824498	0.9761		0.3083857	3.1298716
Other	White	1.0745508	0.8874		0.3972145	2.9068914
**Odds Ratios for Insurance**						
**Level1**	**/Level2**	**Odds Ratio**	**Prob > Chi Sq**		**Lower 95%**	**Upper 95%**
Other	Medicaid	0.570405	0.6841		0.0381792	8.5219704
Other	Private	0.8166524	0.8833		0.0545961	12.215549
Medicaid	Private	1.4317062	0.1559		0.8721007	2.3503969
**Odds Ratios for BMI**						
**Level1**	**/Level2**	**Odds Ratio**	**Prob > Chi Sq**		**Lower 95%**	**Upper 95%**
Overweight	Normal	0.890934	0.7489		0.4392349	1.8071502
Obese	Normal	0.6008151	0.0801		0.3395754	1.0630298
Obese	Overweight	0.6743654	0.3302		0.3051265	1.4904265
**Odds Ratios for Medication Adherence**						
**Level1**	**/Level2**	**Odds Ratio**	**Prob > Chi Sq**		**Lower 95%**	**Upper 95%**
Yes	No	0.6600799	0.1547		0.3725278	1.1695918
**Odds Ratios for Smoking**						
**Level1**	**/Level2**	**Odds Ratio**	**Prob > Chi Sq**		**Lower 95%**	**Upper 95%**
No	Yes	0.5979366	0.1028		0.3223437	1.1091521
**Odds Ratios for Clinic No-Shows Over Previous 24 Months**						
**Level1**	**/Level2**	**Odds Ratio**	**Prob > Chi Sq**		**Lower 95%**	**Upper 95%**
>4	0 (Zero)	2.0889702	0.1096		0.8473165	5.1501372
3–4	0 (Zero)	1.2520752	0.5788		0.5661519	2.7690313
1–2	0 (Zero)	1.4821027	0.1579		0.8584516	2.5588264
>4	1–2	1.4094638	0.4365		0.5937748	3.345693
3–4	1–2	0.8447965	0.6665		0.3922674	1.8193741
>4	3–4	1.6684064	0.3058		0.6263267	4.4442938
**Odds Ratios for Clinic Cancellations Over Previous 24 Months**						
**Level1**	**/Level2**	**Odds Ratio**	**Prob > Chi Sq**		**Lower 95%**	**Upper 95%**
≥11	1–5	1.4152474	0.3763		0.6556921	3.0546736
6–10	1–6	1.7550159	0.0541		0.9902116	3.1105277
≥11	6–10	0.8064015	0.5962		0.3638245	1.7873547
* Statistically significant at the alpha = 0.05 level Note: Normal approximations used for ratio confidence limits effects						
Note: Tests and confidence intervals on odds ratios are Wald based						

## Data Availability

The raw dataset that was analyzed for this study has been included in the Supplementary Materials (Table S4).

## Supplementary Materials

The following supporting information can be downloaded at: https://www.mdpi.com/article/10.3390/ijerph20021508/s1: Table S1: Contingency Table Analysis for DV1; Table S2: Contingency Table Analysis for DV2; Table S3: Contingency Table Analysis for DV3; Table S4: Study Dataset Used for Analysis.
