# Supplementary material for: Effect of Disease Severity, Age of Child, and Clinic No-Shows on Unscheduled Healthcare Use for Childhood Asthma at an Academic Medical Center
Source: Int J Environ Res Public Health. 2023 Jan 13;20(2):1508. doi: 10.3390/ijerph20021508 (PMC9864702; doi:10.3390/ijerph20021508)
Supplement: Supplementary file 1 [file ijerph-20-01508-s001.zip › ijerph-2084602-supplementary/Supplementary Tables.pdf]

Supplementary Tables

Table S1. Contingency Analysis for DV1.

Parameter	Non-Users	Users	Total	%	Fisher's Exact Test p-Value
Individual Demographics (IVs)					
Age: 0-to-<8 years	152	53	205	50%	1.0000
Age: 8-to-<13 years	105	36	141	34%	
Age: 13-to-<17 years	35	12	47	11%	
Age: ≥17 years	13	4	17	4%	
**Total**	**305**	**105**	**410**	**100%**	
Gender: Male	205	66	271	66%	0.4734
Gender: Female	100	39	139	34%	
**Total**	**305**	**105**	**410**	**100%**	
Race: Caucasian	107	28	135	33%	0.2484
Race: African-American	157	66	223	54%	
Race: Hispanic	16	5	21	5%	
Race: Other	25	6	31	8%	
**Total**	**305**	**105**	**410**	**100%**	
Insurance: Medicaid	158	73	231	56%	**0.0042***
Insurance: Private	144	31	175	43%	
Insurance: Other	3	1	4	1%	
**Total**	**305**	**105**	**410**	**100%**	
Individual Risk Factors (IVs)					
Asthma Severity: Intermittent	51	7	58	14%	**<.0001***
Asthma Severity: Mild-Persistent	180	32	212	52%	
Asthma Severity: Moderate-Persistent	65	53	118	29%	
Asthma Severity: Severe-Persistent	9	13	22	5%	
**Total**	**305**	**105**	**410**	**100%**	
BMI: Normal	169	70	239	58%	0.0702
BMI: Overweight	44	15	59	14%	
BMI: Obese	92	20	112	27%	
**Total**	**305**	**105**	**410**	**100%**	
Medication Adherence: Yes	240	80	320	78%	0.5871
Medication Adherence: No	65	25	90	22%	
**Total**	**305**	**105**	**410**	**100%**	
Smoking: Yes	53	19	72	18%	0.8822
Smoking: No	252	86	338	82%	

**Total**	**305**	**105**	**410**	**100%**	
12-Month Clinic No-Shows: 0 (Zero)	270	71	341	83%	**<.0001***
12-Month Clinic No-Shows: 1-to-2	31	22	53	13%	
12-Month Clinic No-Shows: 3-to-4	4	4	8	2%	
12-Month Clinic No-Shows: >4	0	8	8	2%	
**Total**	**305**	**105**	**410**	**100%**	
12-Month Clinic Cancellations: 0 (Zero)	183	35	218	53%	**<.0001***
12-Month Clinic Cancellations: 1-to-5	118	64	182	44%	
12-Month Clinic Cancellations: ≥6	4	6	10	2%	
**Total**	**305**	**105**	**410**	**100%**	

Table S2. Contingency Analysis for DV2.

Parameter	Non-Users	Users	Total	%	Fisher's Exact Test p-Value
Individual Demographics (IVs)					
Age: 0-to-<8 years	138	67	205	50%	0.9361
Age: 8-to-<13 years	95	46	141	34%	
Age: 13-to-<17 years	34	13	47	11%	
Age: ≥17 years	12	5	17	4%	
**Total**	**279**	**131**	**410**	**100%**	
Gender: Male	188	83	271	66%	0.4352
Gender: Female	91	48	139	34%	
**Total**	**279**	**131**	**410**	**100%**	
Race: Caucasian	103	32	135	33%	**0.0331***
Race: African-American	138	85	223	54%	
Race: Hispanic	15	6	21	5%	
Race: Other	23	8	31	8%	
**Total**	**279**	**131**	**410**	**100%**	
Insurance: Medicaid	143	88	231	56%	**0.0064***
Insurance: Private	133	42	175	43%	
Insurance: Other	3	1	4	1%	
**Total**	**279**	**131**	**410**	**100%**	
Individual Risk Factors (IVs)					
Asthma Severity: Intermittent	49	9	58	14%	**<.0001***
Asthma Severity: Mild-Persistent	164	48	212	52%	
Asthma Severity: Moderate-Persistent	58	60	118	29%	
Asthma Severity: Severe-Persistent	8	14	22	5%	
**Total**	**279**	**131**	**410**	**100%**	
BMI: Normal	155	84	239	58%	0.1576
BMI: Overweight	40	19	59	14%	
BMI: Obese	84	28	112	27%	
**Total**	**279**	**131**	**410**	**100%**	
Medication Adherence: Yes	221	99	320	78%	0.4432
Medication Adherence: No	58	32	90	22%	
**Total**	**279**	**131**	**410**	**100%**	
Smoking: Yes	47	25	72	18%	0.5802
Smoking: No	232	106	338	82%	
**Total**	**279**	**131**	**410**	**100%**	
18-Month Clinic No-Shows: 0 (Zero)	246	91	337	82%	**<.0001***
18-Month Clinic No-Shows: 1-to-2	29	24	53	13%	

18-Month Clinic No-Shows: 3-to-4	4	8	12	3%	
18-Month Clinic No-Shows: >4	0	8	8	2%	
**Total**	**279**	**131**	**410**	**100%**	
18-Month Clinic Cancellations: 0 (Zero)	145	42	187	46%	
18-Month Clinic Cancellations: 1-to-5	127	74	201	49%	**<.0001***
18-Month Clinic Cancellations: ≥6	7	15	22	5%	
**Total**	**279**	**131**	**410**	**100%**	

Table S3. Contingency Analysis for DV3.

Parameter	Non-Users	Users	Total	%	Fisher's Exact Test *p*-Value
Individual Demographics (IVs)					
Age: 0-to-<8 years	129	76	205	50%	0.5990
Age: 8-to-<13 years	88	53	141	34%	
Age: 13-to-<17 years	34	13	47	11%	
Age: ≥17 years	12	5	17	4%	
**Total**	**263**	**147**	**410**	**100%**	
Gender: Male	177	94	271	66%	0.5149
Gender: Female	86	53	139	34%	
**Total**	**263**	**147**	**410**	**100%**	
Race: Caucasian	101	34	135	33%	**0.0013***
Race: African-American	124	99	223	54%	
Race: Hispanic	15	6	21	5%	
Race: Other	23	8	31	8%	
**Total**	**263**	**147**	**410**	**100%**	
Insurance: Medicaid	132	99	231	56%	**0.0017***
Insurance: Private	128	47	175	43%	
Insurance: Other	3	1	4	1%	
**Total**	**263**	**147**	**410**	**100%**	
Individual Risk Factors (IVs)					
Asthma Severity: Intermittent	49	9	58	14%	**<.0001***
Asthma Severity: Mild-Persistent	159	53	212	52%	
Asthma Severity: Moderate-Persistent	50	68	118	29%	
Asthma Severity: Severe-Persistent	5	17	22	5%	
**Total**	**263**	**147**	**410**	**100%**	
BMI: Normal	145	94	239	58%	0.1427
BMI: Overweight	38	21	59	14%	
BMI: Obese	80	32	112	27%	
**Total**	**263**	**147**	**410**	**100%**	
Medication Adherence: Yes	208	112	320	78%	0.5346
Medication Adherence: No	55	35	90	22%	
**Total**	**263**	**147**	**410**	**100%**	
Smoking: Yes	42	30	72	18%	0.2800
Smoking: No	221	117	338	82%	
**Total**	**263**	**147**	**410**	**100%**	
24-Month Clinic No-Shows: 0 (Zero)	128	43	171	42%	**<.0001***
24-Month Clinic No-Shows: 1-to-2	92	56	148	36%	

24-Month Clinic No-Shows: 3-to-4	29	21	50	12%	
24-Month Clinic No-Shows: >4	14	27	41	10%	
**Total**	**263**	**147**	**410**	**100%**	
24-Month Clinic Cancellations: 1-to-5	164	70	234	57%	
24-Month Clinic Cancellations: 6-to-10	72	46	118	29%	**0.0028***
24-Month Clinic Cancellations: ≥11	27	31	58	14%	
**Total**	**263**	**147**	**410**	**100%**	
